# A Bayesian algorithm for detecting differentially expressed proteins and its application in breast cancer research

**DOI:** 10.1038/srep30159

**Published:** 2016-07-22

**Authors:** Tapesh Santra, Eleni Ioanna Delatola

**Affiliations:** 1 Systems Biology Ireland, University College Dublin, Belfield, Dublin-4, Ireland

## Abstract

Presence of considerable noise and missing data points make analysis of mass-spectrometry (MS) based proteomic data a challenging task. The missing values in MS data are caused by the inability of MS machines to reliably detect proteins whose abundances fall below the detection limit. We developed a Bayesian algorithm that exploits this knowledge and uses missing data points as a complementary source of information to the observed protein intensities in order to find differentially expressed proteins by analysing MS based proteomic data. We compared its accuracy with many other methods using several simulated datasets. It consistently outperformed other methods. We then used it to analyse proteomic screens of a breast cancer (BC) patient cohort. It revealed large differences between the proteomic landscapes of triple negative and Luminal A, which are the most and least aggressive types of BC. Unexpectedly, majority of these differences could be attributed to the direct transcriptional activity of only seven transcription factors some of which are known to be inactive in triple negative BC. We also identified two new proteins which significantly correlated with the survival of BC patients, and therefore may have potential diagnostic/prognostic values.

MS based proteomics technology can simultaneously measure the abundances of several thousands of proteins in a biological sample. Characterizing changes in protein abundance across groups of samples offers valuable biological insights. However, developing computational methods that can detect such changes is challenging due to considerable noise and variability in MS data. There are three main sources of noise in MS data^1^,^2^. The biggest is the inherent variability of protein samples, which, due to the dynamic nature of proteome, can be greater than variabilities encountered in genomic studies. There are also technological biases related to the method of MS acquisition. Another large source of noise are missing data resulting from the instrument failing to detect weak signals of low-abundance peptides around the detection threshold. This latter issue can lead to typically 10–40% of “missing” measurements in the MS data outputs. Two main strategies have emerged to address this issue. Firstly, missing intensity values set to zero or imputed and subsequently, standard two sample tests are employed to compare peptide abundance between groups^3^,^4^,^5^,^6^. Secondly, the absence-presence data is combined with the intensity values. In this case, the raw mass spectra (m/z ratios) of different peptides are converted into binary absence-presence data where the missing values represent an absence. The binary spectra of each group of samples are then statistically modelled and compared to identify peptides that behave differentially across groups^7^,^8^. However, binary conversion of peptide intensities causes information loss. Therefore, these methods are most effective when each group has a large number of samples (typically >20, e.g. in clinical studies) to make up for the lossy conversion. Most proteomics experiments typically involve three to six samples in each group, thus the above methods may not be appropriate for such data. Additionally, these methods may not be directly applicable to shotgun proteomics experiments which generate protein abundance from mass spectra of peptides, thereby providing a higher level representation of the data.

We addressed these issues by developing a Bayesian method, BDiffProt (Bayesian DIFFerential PROTeomics), that detects differential protein/peptide abundance. It treats missing values as a source of information along with observed intensities, and can be used to analyse different types of proteomics data, even those with small number of samples. We compared its performance and robustness with several other methods using simulated datasets and found it to be more accurate than these methods. We then analysed a proteomic dataset obtained from a cohort of breast cancer (BC) patients using BDiffProt. Our analysis uncovered a transcriptional module, primarily consisting of seven transcription factors, which may be responsible for majority of the differences in proteomic profiles of Luminal A and triple negative breast cancer (TNBC) patients. Changes in the proteomic landscape of TNBC cells had not previously been attributed to direct transcriptional activities of such a small number of transcription factors, and is typically ascribed to aberrant signalling caused by inactive hormone receptors^9^. Additionally, we identified two proteins which had no known association with BC, yet their expressions correlate with survival of BC patients and hence may have prognostic/diagnostic value for BC treatment. Below we describe the details of BDiffProt and its implementation on simulated and real datasets.

## Method

### Formulation

**Figure 1:**
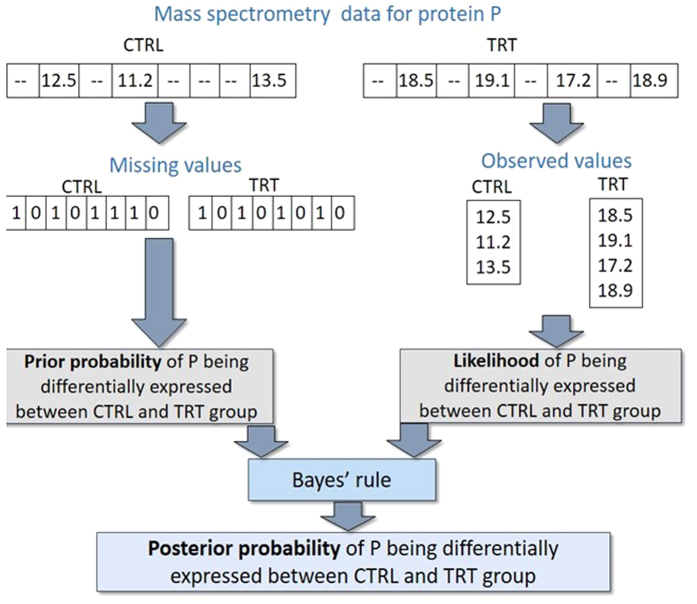
A simplified workflow of the BDiffProt algorithm.

BDiffProt is a Bayesian algorithm which uses experimental data to update prior knowledge/belief about differential protein abundances across groups. It uses the missing values in MS datasets to estimate the prior probability of a protein being differentially expressed between two groups. (Fig. 1). This approach is based on the fact that missing values in MS data typically represent peptides at/below the detection threshold of the MS machines. Hence, a difference in missing values for a particular peptide in multiple samples likely indicates different abundances.

**Figure 2:**
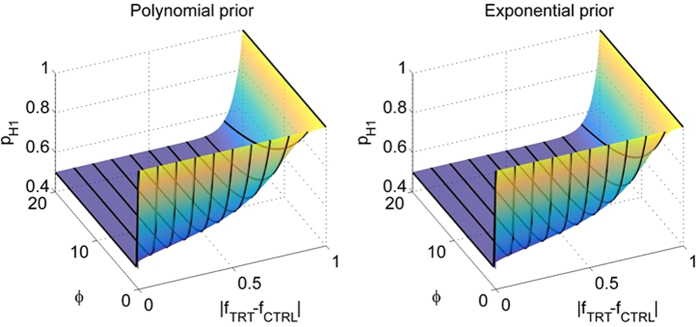
The polynomial (Equation 1) and the exponential (Equation 2) form of the prior probabilities for different values of *ϕ* and |*f*_*CTRL*_ − *f*_*TRT*_|. Each point on the coloured surface represents the value of the polynomial (left panel) and the exponential (right panel) prior for the corresponding values of the coefficient *ϕ* and absolute difference in missing data frequencies |*f*_*CTRL*_ − *f*_*TRT*_|. The black lines on the surfaces represent the values of the polynomial (left panel) and the exponential (right panel) priors at |*f*_*CTRL*_−*f*_*TRT*_| = 0.00001, 0.1, 0.2, 0.3, …, 1 (from left to right).

To elaborate, let us consider a proteomic experiment involving two groups of samples, a control (*CTRL*) and a treatment group (*TRT*). A protein *P* has *n*_*CTRL*_ and *n*_*TRT*_ numbers of observed intensities (
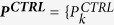
, = 1,.., *n*_*CTRL*_}; 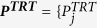, *j* = 1,..., ***n***_*TRT*_}) and ***v***_*CTRL*_ and ***v***_*TRT*_ numbers of missing values in control (*CTRL*) and treatment (*TRT*) groups respectively. We want to find out whether *P* is differentially expressed (hypothesis *H1*) between *CTRL* and *TRT* groups or not (hypothesis *H0*). We first calculate the prior probability (*p*_*H*1_) of *P* being differentially expressed (*H1*) based solely on the frequency of missing values. The frequency of missing values (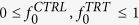) in *CTRL* and *TRT* are given by 
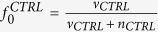
, 
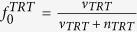
. There are several ways of formulating the prior probability *p*_*H*1_ based on these frequencies, for instance:


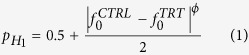



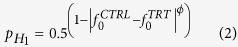


where *ϕ* *>* 0 is a positive real number. The formulations in Eq. 1 and 2 have the following characteristics. If the protein intensities are missing from both groups with equal frequencies (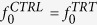), then, H1 and H0 have equal prior probability (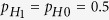), i.e., it is not possible to decide a priori whether the protein is differentially expressed. However, if the protein intensities are missing at a different rates in the two groups, then 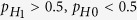, i.e. the protein *P* is likely to be differentially expressed. In extreme cases, where all intensities in one group are missing but none in the other (i.e. either 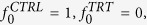 or 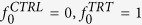), 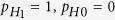 i.e. *P* is believed a priori to be most certainly differentially expressed. The coefficient *ϕ* in Eqs 1,2 determines how sensitive *p*_*H*1_ is on the missing value frequencies 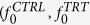) of the *CTRL* and *TRT* groups. Typically, small/large values of *ϕ* makes *p*_*H*1_ highly sensitive/insensitive to these frequencies (Fig. 2) since *ϕ* → 0 and *ϕ* → ∞ implies *p*_*H*1_ → 1 and *p*_*H*1_ → 0.5 respectively, for any 
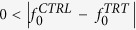
 < 1 (Fig. 2). *p*_*H*1_ is updated based on the observed protein intensities using Bayes’ rule as shown below:


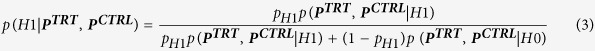


Here, *p*(*H*1 |***P***^***TRT***^, ***P***^***CTRL***^) is updated or posterior probability of hypothesis *H1*, *p*(***P***^***TRT***^, ***P***^***CTRL***^|*H*1) and *p*(***P***^***TRT***^, ***P***^***CTRL***^ |*H*0) are likelihoods of the observed intensities (***P***^***TRT***^, ***P***^***CTRL***^) under hypothesis *H1* and *H0* respectively. The likelihoods are calculated as follows. We assume that, when protein *P* is differentially expressed (*H1*) between *CTRL* and *TRT* groups, the corresponding intensities are normally distributed with means *μ* and *μ* + *τ* respectively, and variance *σ*^2^, 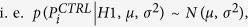 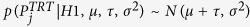
10, where *τ* is the treatment effect. In the opposite case (*H0*), all intensities have the same mean and variance, i.e. 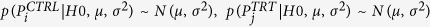
10. *μ*, σ^2^ & *τ* are assumed to have the following prior distributions *μ ~ N*(*μ*_0_, *σ*^2^), *σ*^2^* ~ IG*(*α*, *β*), and τ ~ *N*(τ_0_, *κσ*^2^), where *IG* is inverse gamma distribution, *μ*_0_, *α*, *β*, *τ*_0_ and *κ* are hyper parameters. These assumptions lead to the following form for the likelihood functions (see Supplementary Note 1 for details): h


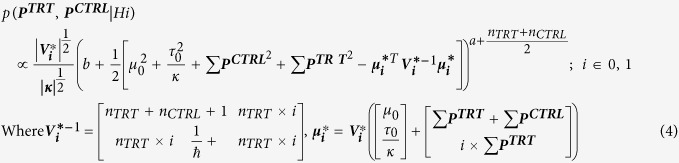


Replacing Eq. (4) in (3), one arrives at the posterior probability (*p*(*H*1|***P***^***CTRL***^, ***P***^***TRT***^)).

### Hyper-parameter optimization

The posterior probability (*p*(*H*1 |***P***^**TRT**^, ***P***^**CTRL**^)) depends on six hyper-parameters *μ*_0_, *α*, *β*,*τ*_0_ *κ* and ϕ whose values are unknown and needs to be estimated. When there are multiple proteins (*P*_*i*_, *i* = 1 … *N*_*p*_) in a dataset, one needs to estimate the values of six hyper parameters (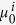, *α*^*i*^, *β*^*i*^, 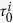 *κ*^*i*^, *ϕ*^*i*^) for each protein (*P*_*i*_). Small sample datasets (typical sample size 3–6), usually do not have enough data for separately estimating the hyper-parameters of each protein. Presence of missing values further complicates this matter. Additionally, estimating large number of hyper-parameters (in this case 6 × *N*_*p*_) can be extremely time consuming. Therefore, we made the simplifying assumption that the hyper-parameters have same values for all proteins within a dataset, i.e. 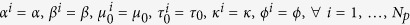 We further assumed that the treatment effects (*τ*_*i*_) can be positive or negative with equal probability and therefore the mean treatment effect *τ*_0_ = 0. The remaining five hyper parameters (*μ*_0_, *α*, *β*, *κ*, ϕ) are estimated to maximize the marginal likelihood function:


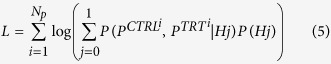


We used the active set algorithm, implemented in MATLAB’s fmincon function for optimization (see http://uk.mathworks.com/help/optim/ug/constrained-nonlinear-ptimization-algorithms.html#brnox01 for details). Data was standardized before optimization to ensure that some of the above assumptions are at-least approximately met. A protein *P*_*i*_ is standardized by subtracting the sample mean 
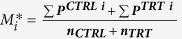
 from its measurements and dividing the resulting values by the sample standard deviation 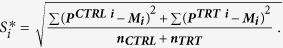 The optimal values of hyper-parameters were used to calculate the posterior probabilities 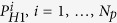 of proteins *P*^i^, *i* = 1, …, *N*_*p*_ being differentially expressed.

### False discovery rate control

A Bayesian multiple testing criterion^11^ was applied to control the false discovery rate (FDR). The objective of the above is to determine a threshold probability 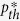, which ensures that there are *α*% falsely discovered proteins among those with higher posterior probabilities than *p*_th_^*^. To find 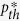, expected FDRs at different values (*p*_*th*_) of threshold probabilities are calculated using the following formulation:


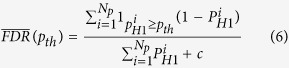


Here, *N*_*p*_ is the total number of proteins, 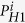 is posterior probability of hypothesis *H1* for the *i*^*th*^ protein, 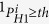 is 1 when 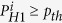 and 0 otherwise, and *c* is an offset parameter. For a given FDR level *α*, the optimal threshold 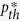 is defined as :


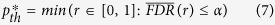


## Results

### Simulation Study

**Figure 3:**
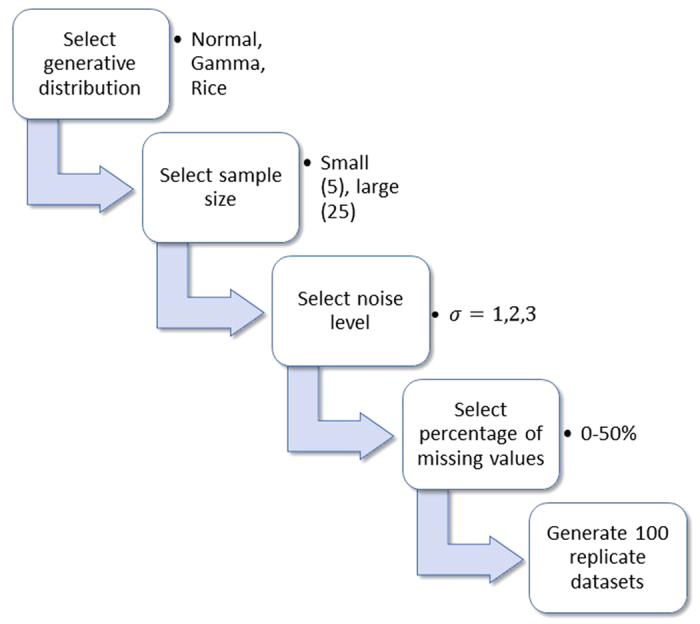
Workflow of the simulated data generation process.

We performed an extensive simulation study to evaluate the performance of BDiffProt and compare its accuracy with other methods which are widely used in proteomics data analysis. For this purpose, we simulated a large number of datasets with different levels of noise (low, medium, high), percentages of missing values (0%, 10%, 20%, 30%, 40%, 50%), numbers of samples (small sample = 5, large sample = 25) in each experimental condition and the types of generative distributions (Normal, Gamma and Rician). See Fig. 3 for the data simulation workflow. Normally distributed datasets broadly reflect statistical properties of typical high-throughput proteomic data^12^,^13^, whereas Rician and Gamma distributed datasets allow evaluation of BDiffProt’s performance on non-normal data which violate it’s assumption of data Normality.

In the Normally distributed data, the log intensities of the *i*^*th*^ protein (*P*_*i*_) were generated by sampling from *N*(*μ*_*i*_,*σ*^2^) and 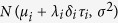 for the *CTRL* and *TRT* groups respectively. Here, *μ*_*i*_ is the mean log-intensity of the *CTRL* group and was randomly generated by sampling from *N*(15,3), *λ*_*i*_ indicates whether *P*_*i*_ is differentially expressed (*λ*_*i*_ = 1) or not (*λ*_*i*_ = 0) and was assigned 1 or 0 with equal probability. *δ*_*i*_ indicates whether *P*_*i*_ has higher (*δ*_*i*_ = 1) or lower (*δ*_*i*_ = −1) intensities in *TRT* than *CTRL* and was randomly assigned 1 or −1 with equal probability. *τ*_*i*_ is the treatment effect and was sampled from the following gamma distribution Γ(10,0.5). *σ*^2^ is the noise variance.

In the Gamma distributed datasets, the log intensities of the *i*^*th*^ protein (*P*_*i*_) were generated by sampling from the Gamma distributions Γ(*a*_*i*_, *b*_*i*_) and 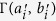 for the *CTRL* and *TRT* groups respectively. Here, *a*_*i*_, 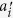 are shape and 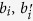 are scale parameters. The values of these parameters were calculated as follows: 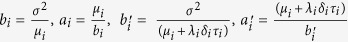. Here *μ*_*i*_ is the mean log-intensity of protein *P*_*i*_ in the *CTRL* group and was generated by sampling from Γ(56.25, 0.2667) which ensures that *μ*_*i*_ has mean and standard deviations of 15 and 2 respectively. *λ*_*i*_, *δ*_*i*_, *τ*_*i*_ were generated as described in the previous case.

In Rician datasets, the log intensities of the *i*^*th*^ protein (*P*_*i*_) were generated by sampling from the Rice distributions R(*μ*_*i*_, *σ*) and *R*(*μ*_*i*_ + *λ*_*i*_ *δ*_*i*_ *τ*_*i*_, *σ*) for the *CTRL* and *TRT* groups respectively. Here *μ*_*i*_ was generated by sampling from the Rice distribution *R*(15,*σ*) and *λ*_*i*_ *δ*_*i*_, *τ*_*i*_ were generated as described before.

Each dataset contains log-intensities of 1000 proteins measured in two conditions, control (*CTRL*) and treatment (*TRT*). Mimicking the behaviour of MS machines, we introduced missing values in each dataset by replacing the smallest log-intensity values by zero. We introduces different levels of noise (*σ* = 1,2,3) and missing values (0%, 10%, 20%, 30%, 40%, 50%) in the datasets. At *σ* ≥ 4, the treatment effects (*δ*_*i*_*τ*_*i*_) becomes largely indistinguishable from noise since the average magnitude of the treatment effect is approximately 10 × 0.5 = 5. Therefore we restricted our analysis to only three levels of noise *σ = *1,2,3. Some of the datasets had small sample sizes (5 samples per experimental condition) and some had relatively large sample sizes (25 samples per experimental condition). The number of samples were chosen to mimic cell line and tissue based proteomic data-sets which typically have 3–6 and >15 samples per condition respectively. 100 replicate data sets were generated for each level of noise, missing values, sample sizes and generative distributions resulting in 3600 Normal, Gamma and Rician datasets each (total 10800 datasets).

BDiffProt with two different prior settings (Equations 1,2) were applied to each of these datasets, and the posterior probabilities of differential expression were estimated for each protein. Subsequently, Area under the Receiver Operating Characteristic curve (AUROC) was used for performance evaluation^14^. ROC curve was calculated by evaluating true and false positive rates for increasing values of the threshold probabilities (*p*_*th*_) which separate the differentially expressed proteins (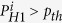) from the rest, and then integrating the true positive rates with respect to false positive rates^14^ across the full range of plausible values of the threshold probabilities (*p*_*th*_ ∈ [0,1]). AUROC can be between 0 and 1, and the closer it is to 1 the better the performance, with AUROC = 1 being the ideal case. Mean and standard deviations of the AUROC values calculated for 100 replicate datasets of each category were used to indicate the accuracy of BDiffProt and the corresponding confidence interval for that category.

**Figure 4:**
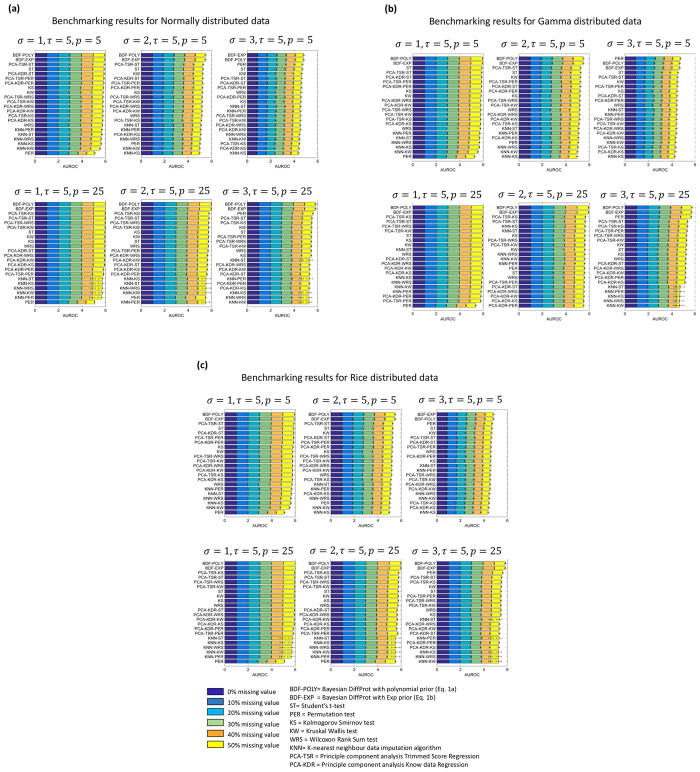
Benchmarking BDiffProt algorithm on simulated datasets. (**a–c**) Benchmarking results for Normal, Gamma and Rice distributed datasets respectively. Mean AUROCs and the corresponding standard deviations are represented by horizontal stacked bar charts and the error-bars. Average AUROCs at different levels of missing values are represented by different colours. In each chart, top overall performers across all missing value levels are displayed on top, and the worst performers are displayed at the bottom.

The performance of BDiffProt was compared with several other methods. Common methods of finding differentially expressed proteins involve missing value imputation followed by hypothesis tests. We selected five different hypothesis tests, t-test^15^, Wilcoxon Rank Sum test (WRS) test^16^, Kruskal Wallis (KW) test^17^, Kolmogorov Smirnov (KS) test^18^ and Permutation (PER) test^19^ and three missing value imputation methods, k-nearest neighbour (KNN)^20^, Principal Component Analysis with known data regression (PCA-KDR)^21^, Principal Component Analysis with trimmed scores regression (PCA-TSR) for performance comparison^21^ since these are some of the most commonly used methods in proteomics data analysis studies^22^,^23^,^24^,^25^,^26^,^27^. Hypothesis tests were implemented individually or in combination with one of the three imputation methods. The performances of these methods were evaluated in the same way as BDiffProt. The results (Fig. 4) suggest that BDiffProt outperformed other methods in almost all cases. Interestingly, BDiffProt, which assumes data Normality, outperformed WRS, KW, KS and PER which do not make such assumption, in non-Normal (Gamma and Rician) datasets. This may be due to the fact that most small sample datasets satisfy approximate normality unless its distribution has long tails^28^,^29^,^30^,^31^,^32^,^33^,^34^,^35^ which is not the case for Gamma and Rician distribution. Additionally, non-parametric methods such as WRS, KW, KS and PER suffer from lack of power when sample sizes are small^28^,^29^,^30^,^31^,^32^,^33^,^34^,^35^. Finally, BDiffProt’s capability of extracting valuable information from missing values further enhanced its accuracy. Indeed, the accuracy of most methods dropped significantly with increasing levels of missing values, whereas BDiffProt suffered relatively small performance drops (Fig. 4).

There were no significant difference between the performance of BDiffProt with polynomial and exponential priors. Most other methods performed the worst when applied in combination with KNN based missing value imputation methods, whereas PCA based imputation methods resulted in better performances. PER performed the best among other methods when data had high level of noise. T-test consistently performed well among other methods.

Encouraged by the superior performance of BDiffProt we used it to analyse a proteomic dataset obtained from a cohort of Breast Cancer patients^36^. Below we discuss the results of our analysis.

### Breast Cancer Data

Breast cancer has several subtypes that differ in aggressiveness and clinical outcome. The most common subtypes are Luminal A, Luminal B, Triple negative/basal like (TNBC) and HER2 positive^37^. These are characterized mainly by the status of three receptors (Estrogen, Progesteron and HER2 Receptor) and a gene which regulates proliferation (Ki67).

- Luminal A is Estrogen (ER) and/Progesteron (PR) positive (overexpressed/highly active), HER2 and Ki67 negative (low abundance/inactive). Survival of Luminal A patients were also recently shown to correlate with p53^38^.
- Luminal B is ER and/or PR positive, HER2 positive or HER2 negative but Ki67 positive.
- HER2 positive is ER, PR negative, but HER2 positive
- TNBC is ER, PR and HER2 negative

Currently, there is a concerted effort by international consortiums (e.g. TCGA http://cancergenome.nih.gov/) to characterize the molecular differences between different BC subtypes beyond their receptor and Ki67 status. As part of this effort, proteome-wide protein abundance data of a cohort of BC patients was recently made available by the TCGA consortium^36^ from the CTPAC portal (https://cptac-data-portal.georgetown.edu/cptac/s/S015). The dataset consists of relative abundances of 10599 proteins in 105 BC tumour samples along with their pathological subtype characterizations. Protein expression was measured using iTRAQ (isobaric Tags for Relative and Absolute Quantification) protein quantification methods. We used BDiffProt with polynomial prior (Equation 1) to compare the abundances of different proteins across BC subtypes. Firstly, we checked whether the above dataset satisfies BDiffProt’s data Normality assumption. We separately used four different hypothesis tests, Kolmogorov-Smirnov^39^ (KS), Lilliefors^40^ (LF), Shapiro-Wilkies^41^ (SW) and Anderson-Darling^42^ (AD) to check whether the observed expressions of each protein is Normally distributed in each BC subtype. Each test produces a p-value which indicates how well the data supports the hypothesis that the data is Normally distributed. Following common practice, we assumed that the above hypothesis can be rejected with confidence if the corresponding p-value is less than 0.05. Normality tests were performed if at-least four observed expressions were available. The Normality hypothesis could not be rejected for 99.0364%, 76.8822%, 79.5845% and 83.3484% of cases when KS, SW, AD and LF tests were used respectively, suggesting that the majority of the data is likely to be at least approximately Normal.

We then divided the protein expressions in four groups, Luminal A, Luminal B, Her 2 positive and Basal. Since the patient cohort does not include any normal person who do not have BC, samples from Luminal A were considered to be the control group as it is the less aggressive of the four subtypes. The proteomic profiles of Luminal B, Her 2 positive and Basal BC patients were compared with those of the Luminal A patients using BDiffProt, followed by FDR correction (Eq. 7). Surprisingly, at 1% FDR, we found only six differentially expressed proteins (ACOT7, BDH2, DSCC7, HSP90AB1, LMNA, MYOF) between Luminal A which is a HER2 negative BC subtype, and Luminal B which can be either HER2 negative or occasionally HER2 positive^43^. However, Luminal A and Luminal B are both ER positive, low grade and known to have similar molecular characteristics^44^. Additionally, 70% of Luminal B patients in the above cohort are HER2 negative and only 30% have HER2 positive mutations. This implies that the majority of Lumina A and Luminal B patients in this cohort share similar receptor status, which may explain why we did not find any significant difference between the proteomics profiles of these two groups of patients.

**Figure 5:**
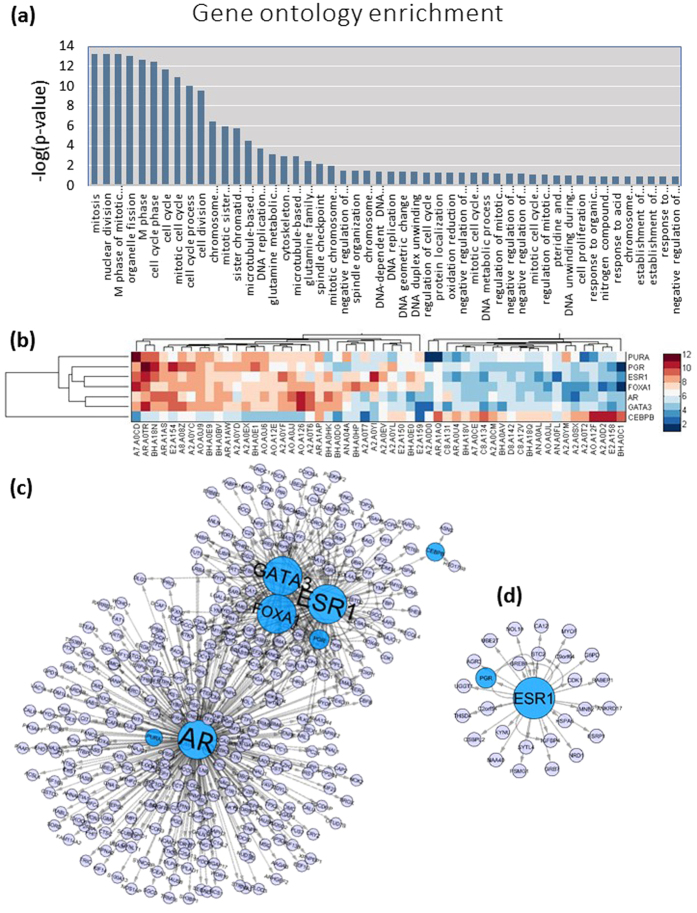
Ontology and transcriptional program analysis of the differentially expressed proteins in TNBC and HER2 positive BC patients. (**a**) Over represented gene ontology terms for differentially expressed proteins in TNBC cells. (**b**) Heatmap showing expressions of the seven TFs in Luminal A and TNBC patients. (**c,d**) Transcriptional modules identified in TNBC and HER2 positive cells.

On the other hand, 705 and 163 proteins were found to be differentially expressed in the triple negative and HER2 positive patients respectively (Supplementary data 1,2). We used PantherDB^45^ to identify statistically overrepresented gene and pathway ontology annotations in these proteins. The gene ontology analysis suggested that the differentially expressed proteins in triple negative BC (TNBC) patients participate in several biological processes, e.g. cell cycle, cytoskeleton organization, DNA replication, metabolism etc. (Fig. 5a), which are directly affected by cancer and metastasis. The pathway ontology analysis found only two significantly overrepresented pathways (Serine glycine biosynthesis and PLP biosynthesis) among the proteins which were differentially expressed between Luminal A and TNBC. No enriched pathways were found among proteins which were differentially expressed between Luminal A and Her positive/Luminal B. This suggests that the expression of proteins belonging to common cancer related pathways did not collectively change between Luminal A and other BC subtypes. This may seem surprising since many signalling pathways are regulated by the hormone receptors, the status of which characterize these subtypes^46^. However, these pathways operate via post translational modifications (e.g. phosphorylation/de-phosphorylation/acetylation/cleavage etc.) which have little effect on protein abundances. Since we compared protein abundances, it is not surprising that the differential activities of signalling pathways downstream to ER/PR/HER2 receptors were not apparent in our study. The differences in protein abundances may instead be consequences of other biological processes such as transcriptional regulation which have significant influence on protein abundance.

We looked at the transcriptional programs of the differentially expressed proteins in triple negative and HER2 positive subtypes. We used HTRIdb^47^ database to identify known transcription regulators of these proteins. Interestingly, we found that more than half (361 out of 705) of the proteins which are differentially expressed in TNBC are part of a transcription regulatory module consisting of seven transcription factors (TFs), Androgen Receptor (AR), Estrogen receptor (ESR1), Progesteron receptor (PGR), FOXA1, GATA3, PURA, CEBPB and their known targets (Supplementary data 3). A heatmap (Fig. 5b) of the expression levels of these TFs further revealed that all of them except CEBPB have significantly lower expression levels in TNBC compared to Luminal A patients, whereas the opposite is true for CEBPB. It is logical to assume that the collective differential expressions of these TFs and their known targets between Luminal A and TNBC/HER2-positive patients may be causally related. For instance, ER is a known transcriptional regulator of FOXA1, and both were found to be relatively highly expressed in Lumina A patients but have much lower expression levels in TNBC patients. Therefore, it can be assumed that the lower expression levels of FOXA1 in TNBC patients is, at least in part, due to the absence of ER in these patients. The same applies to each TF-target pair shown in Fig. 5c,d. This implies that the differences in the proteomics landscapes of different BC subtypes is at least partly caused by the lack/absence of transcriptional regulation by hormone receptors which are not expressed in these patients.

It is commonly believed that inactivity of Estrogen, Progesteron and HER2 receptors indirectly alters transcriptional program in TNBC cells by altering downstream signalling^9^. However, our analysis suggests that the majority of differentially expressed proteins are known direct transcriptional targets of these receptors, as opposed to being indirectly regulated by these receptors via signalling pathways. We further investigated whether the above transcriptional module has any known association with TNBC. Besides ESR1 and PGR which are well known markers of TNBC^37^, AR, FOXA1 GATA3, CEBPB were recently shown to play major roles in proliferation and migration of the same BC subtype^48^,^49^,^50^,^51^. PURA does not currently have a known association with BC. However, it is a known transcription regulator of AR which is the biggest transcriptional hub among the differentially expressed proteins in TNBC. Regulators of large hubs in complex networks are known to play influential roles in the determining the dynamics of the network^52^. Therefore, the low expression of PURA in TNBC patients may have significant influence in their transcriptional programs, ultimately affecting their survival. Analysis of survival data of a different breast cancer patient cohort^53^ using Kaplan Meier plot (kmplot) revealed that PURA has statistically significant (p-value = 1.7 × 10^−10^) association with relapse free survival of BC patients (Supplementary Fig. S1). This makes PURA a potentially new biomarker for TNBC patients.

We did not find any significantly enriched gene/pathway ontology for the proteins that were differentially expressed in HER2 positive patients. However, a few proteins (ERBB2, IRS1, Integrins, GSK3B etc.) in this list are known to participate in several cancer related pathways such as ERBB signalling, Insulin signalling, Angiogenesis pathway, Ras pathway etc. A transcriptional module involving ESR1 and its known targets was also identified among these proteins (Supplementary data 4). Some of the proteins in this module also correlate with BC patient survival. For instance, SYTL4, which binds to Rab GTPases (http://www.genecards.org/cgi-bin/carddisp.pl?gene=SYTL4), has no known association with breast cancer, but it was identified to be differentially expressed in both HER2 positive and triple negative tumours. A Kaplan Meier plot revealed that it has statistically significant correlation with relapse free survival of BC patients (Supplementary Fig. S2). Therefore, it can also have therapeutic and diagnostic value in BC treatments.

## Discussion

We are living in the age of big data. Enormous amount of data is being produced every day in all walks of life. Our capability of analysing these data to extract valuable information has fallen far behind that of data generation. This is further abated by the quality of data being produced. In biology, data quality is affected by several factors ranging from manual error by experimentalists, to random and systematic error imposed by the data acquisition systems. To make the most of biological data, it is necessary to separate systematic errors from random noise, and exploit the knowledge of the underlying mechanisms that causes such errors to our advantage. In this paper, we developed a Bayesian algorithm BDiffProt that exploits a technical limitation of MS machines to identify differentially expressed proteins in MS data with increased accuracy. We demonstrated its superior performance using simulated data, and its usefulness using real experimental data obtained from breast cancer patients.

However, BDiffProt assumes that the observed protein intensities are normally distributed in each experimental condition, and thus, it is recommended that, appropriate statistical tests should be performed to determine whether a dataset satisfies the normality assumption before applying BDiffProt. This is especially true when the dataset have relatively large (typically >50) number of samples per condition. However, the vast majority of proteomics datasets have relatively small number of samples. We have shown in this study that BDiffProt performs well on non normal datasets when the sample size is relatively small as long as the data does not have heavy tailed distributions.

Finally, the performance of BDiffProt can be further improved by exploiting other sources of systematic errors and existing prior knowledgebase. For instance, prior knowledge of protein protein interactions and genetic interactions may be exploited to extract valuable information from seemingly noisy proteomics data. We shall address some of these issues in the next iteration of BDiffProt.

### Availability and Implementation

BDiffProt was implemented in MATLAB and can be found in: https://github.com/SBIUCD/BDiffProt.git

## Additional Information

**How to cite this article**: Santra, T. and Delatola, E. I. A Bayesian algorithm for detecting differentially expressed proteins and its application in breast cancer research. *Sci. Rep.* **6**, 30159; doi: 10.1038/srep30159 (2016).

## Supplementary Material

Supplementary Information
