# Supplementary material for: A Bayesian algorithm for detecting differentially expressed proteins and its application in breast cancer research
Source: Sci Rep. 2016 Jul 22;6:30159. doi: 10.1038/srep30159 (PMC4957118; doi:10.1038/srep30159)
Supplement: Supplementary Information [file srep30159-s1.pdf]

Supplementary note 1: A Bayesian algorithm for detecting differentially expressed proteins and its application in breast cancer research

Tapesh Santra[1*], Eleni Ioanna Delatola[1]

[1]Systems Biology Ireland, University College Dublin, Belfield, Dublin-4, Ireland

Eleni Ioanna Delatola is postodoctoral researcher at Systems Biology Ireland.

Tapesh Santra is junior group leader at Systems Biology Ireland.

[*] Corresponding author: tapesh.santra@ucd.ie

The statistical model of BDiffProt can be represented in the following linear form:

$$\mathbf{Y} = \mathbf{X_{Hi}}\boldsymbol{\beta} + \epsilon,\ i = 0,1 \tag{1}$$

where $\mathbf{Y}$ is a $n \times 1$ vector ($n = n_{CTRL} + n_{TRT}$) that contains the protein intensities [$\boldsymbol{P^{CTRL}}$; $\boldsymbol{P^{TRT}}$] in the *CTRL* and *TRT* groups respectively. $\boldsymbol{X_{Hi}}, i = 0,1$ are $n \times 2$ input matrices in the null (*H0*) and alternative hypothesis (*H1*) respectively and have the following forms:

$$X_{H0} = [\mathbf{1}_{n\times1}\ \mathbf{0}_{n\times1}] \qquad \text{(a)}$$

$$X_{H1} = [\mathbf{1}_{n\times1}\ \boldsymbol{I_H}],\ \ \boldsymbol{I_H} = \begin{bmatrix}\mathbf{0}_{n_{CTRL}\times1}\\ \mathbf{1}_{n_{TRT}\times1}\end{bmatrix}_{n\times1} \qquad \text{(b)} \tag{2}$$

$\boldsymbol{\beta}$ is a 2×1 vector of DiffProt parameters, i.e. $\boldsymbol{\beta} = \begin{bmatrix}\mu\\ \tau\end{bmatrix}$, and ϵ is Gaussian noise with zero mean and standard deviation σ, i.e. $\epsilon \sim N(0, \sigma^2)$. $\boldsymbol{\beta}$ has the following multivariate Normal prior distribution:

$$\boldsymbol{\beta} \sim \boldsymbol{N}(\boldsymbol{\mu_\beta}, \sigma^2\mathbf{V_\beta}),\ \ \boldsymbol{\mu_\beta} = \begin{bmatrix}\mu_0\\ \tau_0\end{bmatrix}, \boldsymbol{V_\beta} = \begin{bmatrix}1 & 0\\ 0 & \kappa\end{bmatrix}, \kappa > 0 \tag{3}$$

And finally, the noise variance σ^2 has inverse gamma distribution with parameters a and b respectively:

$$\sigma^2 \sim IG(a, b) \tag{4}$$

The above assumptions lead to the following likelihood function which can be used to calculate the probability of the observed protein intensities ($\boldsymbol{Y}$) in the context of a certain hypothesis ($Hi, i = 0,1$) when the parameters $\boldsymbol{\beta}$ and the noise variance σ^2 is known.

$$P(\boldsymbol{Y}|\boldsymbol{\beta}, \sigma^2, Hi) = N(\boldsymbol{X_{Hi}}\boldsymbol{\beta}, \sigma^2) \tag{5}$$

The marginal likelihood $P(\boldsymbol{Y}|Hi)$ which represents the average probability of observing the protein intensities in the contexts of hypothesis Hi across all possible values of $\boldsymbol{\beta}, \sigma^2$ can be calculated from Eq. 5 as shown below.

$$P(\boldsymbol{Y}|Hi) = \iint N(\boldsymbol{X_{Hi}\beta}, \sigma^2)\,\boldsymbol{N}(\boldsymbol{\mu_\beta}, \boldsymbol{\Sigma_\beta})IG(a,b)d\boldsymbol{\beta}\,d\sigma^2$$
$$= \frac{1}{(2\pi\sigma^2)^{\frac{n+p}{2}}|\boldsymbol{V_\beta}|^{\frac{1}{2}}}\int\int \exp\left(-\frac{1}{2\sigma^2}\left\{(\boldsymbol{Y}-\boldsymbol{X_{Hi}\beta})^T(\boldsymbol{Y}-\boldsymbol{X_{Hi}\beta})\right.\right.$$
$$\left.\left.+\left(\boldsymbol{\beta}-\boldsymbol{\mu_\beta}\right)^{\boldsymbol{T}}\boldsymbol{V_\beta^{-1}}\left(\boldsymbol{\beta}-\boldsymbol{\mu_\beta}\right)\right\}\right)d\boldsymbol{\beta}\times\frac{b^a}{\Gamma(a)}\left(\frac{1}{\sigma^2}\right)^{a+1}\exp\left(-\frac{b}{\sigma^2}\right)d\sigma$$
$$= \frac{b^a}{(2\pi\sigma^2)^{\frac{n}{2}}\left|\boldsymbol{I}+\boldsymbol{X_{Hi}V_\beta X_{Hi}^T}\right|^{\frac{1}{2}}\Gamma(a)}\int\left(\frac{1}{\sigma^2}\right)^{a+1}\exp\left(-\frac{1}{\sigma^2}\left\{b\right.\right.$$
$$\left.\left.+\frac{1}{2}\left(\boldsymbol{Y}-\boldsymbol{X_{Hi}\mu_\beta}\right)^T\left(\boldsymbol{I}+\boldsymbol{X_{Hi}V_\beta X_{Hi}^T}\right)^{-1}\left(\boldsymbol{Y}-\boldsymbol{X_{Hi}\mu_\beta}\right)\right\}\right)d\sigma$$
$$= MVSt_{2a}\left(\boldsymbol{X_{Hi}\mu_\beta}, \frac{b}{a}\left(\boldsymbol{I}+\boldsymbol{X_{Hi}V_\beta X_{Hi}^T}\right)\right)$$
$$= \frac{b^a\Gamma\left(a+\frac{n}{2}\right)\sqrt{|\boldsymbol{V_i^*}|}}{(2\pi)^{\frac{n}{2}}\Gamma(a)\sqrt{|\boldsymbol{V_\beta}|}}\left[b+\frac{1}{2}\left\{\boldsymbol{\mu_\beta^T V_\beta^{-1}\mu_\beta}+\boldsymbol{Y^TY}-\boldsymbol{\mu_i^{*T}V_i^{*-1}\mu_i^*}\right\}\right]^{-\left(a+\frac{n}{2}\right)}$$

Here $MVSt_{2a}$ represents a multivariate student t distribution,

$$\boldsymbol{V_i^{*}}^{-1} = \left(\boldsymbol{V_\beta^{-1}}+\boldsymbol{X_{Hi}^TX_{Hi}}\right) = \begin{bmatrix} n+1 & n_{TRT}\times i \\ n_{TRT}\times i & \frac{1}{\kappa}+n_{TRT}\times i\end{bmatrix},$$

$$\boldsymbol{\mu_i^*} = \boldsymbol{V_i^*}\left(\boldsymbol{V_\beta^{-1}\mu_\beta}+\boldsymbol{X_{Hi}^TY}\right) = \boldsymbol{V_i^*}\left(\begin{bmatrix}\mu_0 \\ \frac{\tau_0}{\kappa}\end{bmatrix}+\begin{bmatrix}\sum\boldsymbol{Y} \\ i\times\sum\boldsymbol{P^{TRT}}\end{bmatrix}\right), \quad (6)$$

Replacing $\boldsymbol{\mu_\beta}, \boldsymbol{V_\beta}$ from Eq. 3 into Eq. 6 we arrive at:

$$p(\boldsymbol{Y}|Hi) \propto \frac{|\boldsymbol{V_i^*}|^{\frac{1}{2}}}{|\boldsymbol{\kappa}|^{\frac{1}{2}}}\left(b+\frac{1}{2}\left[\mu_0^2+\frac{\tau_0^2}{\kappa}+\boldsymbol{Y^TY}-\boldsymbol{\mu_i^{*T}V_i^{*-1}\mu_i^*}\right]\right)^{a+\frac{n}{2}};\ i \in 0,1 \quad (7)$$

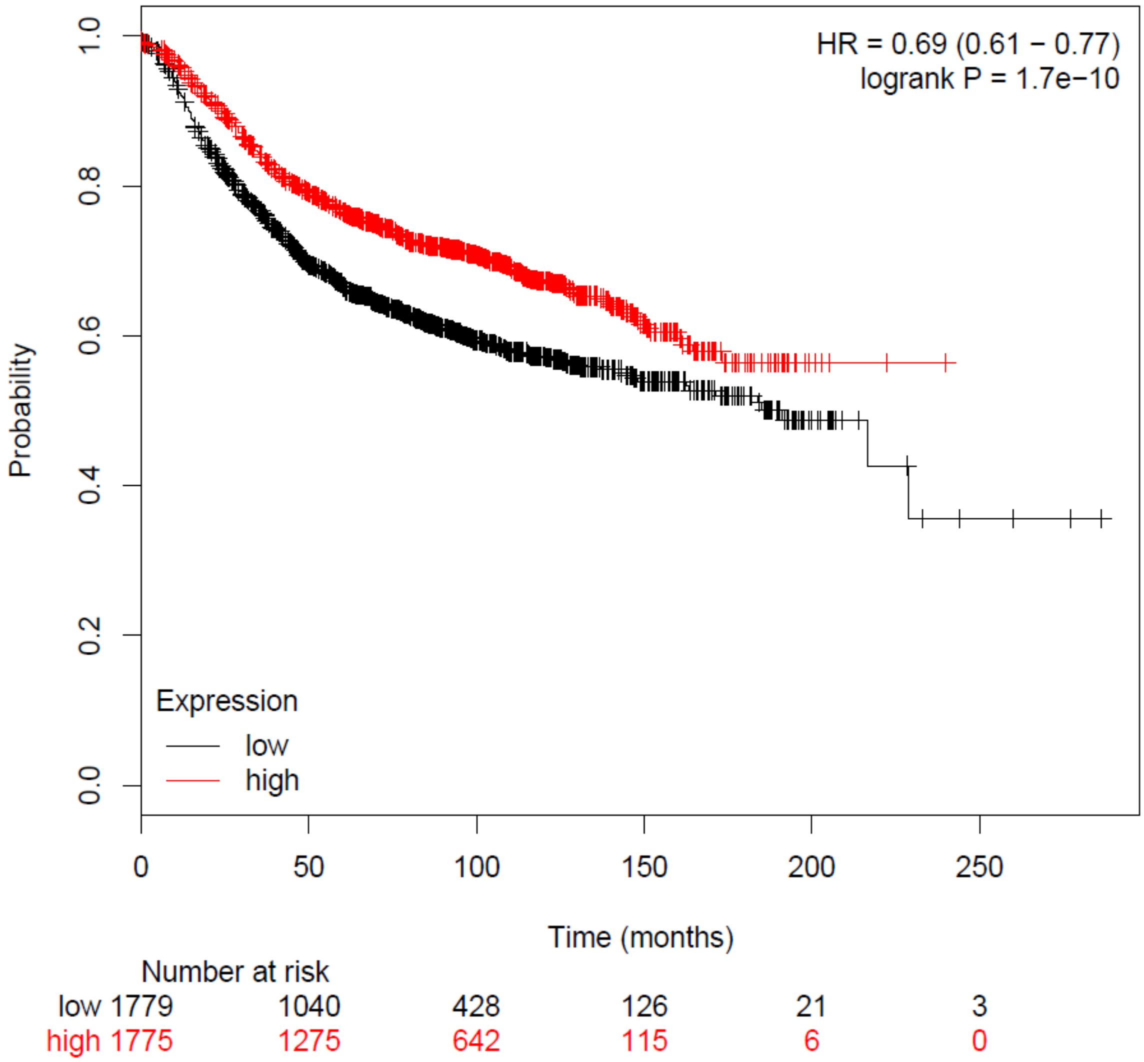

Figure S1: Kaplan Meier plot for PURA. X-axis represents time in months, Y-axis represents probability of survival.

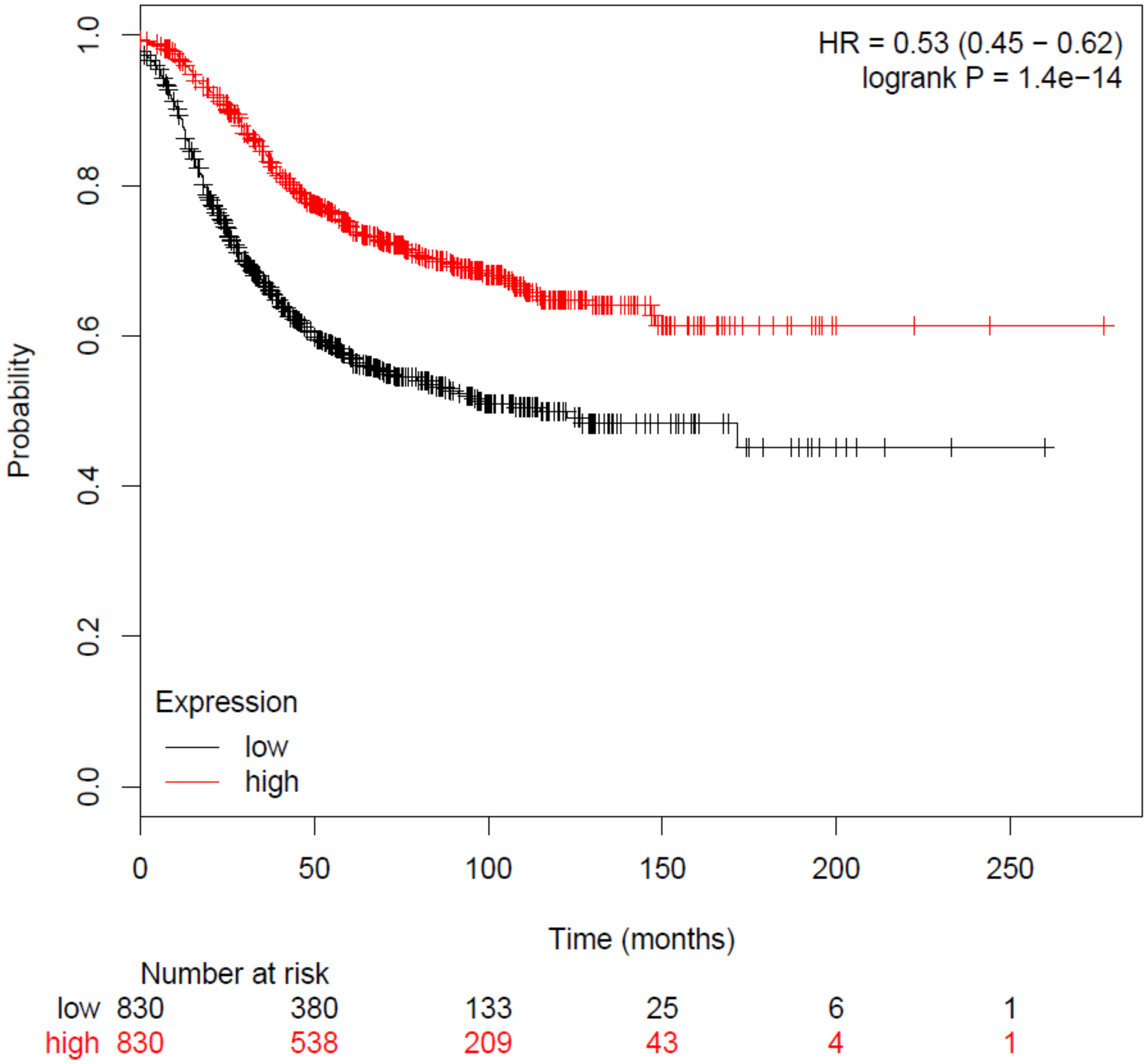

Figure S2: Kaplan Meier plot for SYTL4. X-axis represents time in months, Y-axis represents probability of survival.

Supplementary Data 1: List of differentially expressed proteins between Luminal A and Basal subtypes.

A2ML1,AAGAB,AARS,AARSD1,ABAT,ACADSB,ACAP1,ACSF2,ACSL4,ACTN4,ACY1,ADSL,AFTPH,AGR2,AGR3,AHCYL1,AHDC1,AHNAK,AIF1L,AKR7A3,AKT2,ALDH6A1,ANKRD17,ANKRD50,ANKZF1,ANLN,ANP32E,ANXA9,AP1G2,AP3B1,AP3D1,APBB2,APPL2,AR,ARFIP1,ARFIP2,ARHGAP17,ARHGAP35,ARHGEF1,ARHGEF2,ARL3,ASAH1,ASL,ASMTL,ASNS,ASPN,ASS1,ASUN,ATG3,ATP8B1,AURKA,BAG1,BAZ1B,BCAM,BCAS1,BCAS3,BCAT2,BCL2,BEND3,BICD2,BIRC5,BLVRA,BOD1,BOP1,BPI,BROX,BSPRY,C12orf10,C16orf13,C1orf27,C3orf58,C6orf211,C9orf142,C9orf64,C9orf89,CA12,CACYBP,CALR,CALU,CAP2,CAPN13,CASP6,CBFB,CBR4,CBS,CBX2,CCDC134,C

DC123,CDC20,CDC42BPG,CDCA2,CDCA5,CDH3,CDK1,CDK6,CDYL2,CEBPB,CECR5,CELSR1,CENPE,CENPF,CEP170,CETN3,CHCHD5,CHORDC1,CHTF18,CIB1,CIRH1A,CIT,CKAP2,CKS1B,CLK3,CLSTN2,CLUH,CMA1,CMBL,COASY,COG1,COG2,COL4A3BP,COLGALT1,COPRS,COPS4,COPS7A,CORO2A,CPA3,CRABP1,CRAT,CRBN,CRIP1,CROCC,CROT,CRYZ,CST3,CTPS1,CTSC,CUL3,CXXC5,CYB5A,CYB5R1,DAK,DCAF11,DCXR,DDB2,DDX18,DDX19A,DDX21,DDX39A,DDX47,DDX49,DENND2D,DESI1,DHRS3,DIAPH2,DLG3,DLGAP5,DMD,DNAJB11,DNAJC1,DNAJC12,DNAJC3,DNAL1,DNALI1,DNMT3A,DNPEP,DOCK1,DOK2,DRG1,DSC2,DSCC1,DYNLL2,DYNLT1,ECI2,ECT2,EDARADD,EDC4,EEF1G,EEF2K,EGFR,EHBP1,EHD1,EMC2,EMC7,EML2,EOGT,EPB41L5,EPHB3,EPM2AIP1,ERBB3,ERCC6L,ERGIC1,ERP44,ESR1,ESRP1,ETFDH,EVL,EXOC2,EXOC6,EXTL2,EZH2,FAAH,FABP5,FABP7,FAH,FAM102A,FAM114A2,FAM120A,FAM172A,FAM198B,FAM213B,FAM63A,FAM98A,FAM98C,FANCD2,FAT1,FBP1,FBXO38,FERMT1,FGD3,FHIT,FLII,FLNB,FLYWCH2,FOLR1,FOXA1,FOXP1,FPGT,FSCN1,FUT8,FYCO1,GABARAPL2,GALNT6,GALNT7,GAMT,GARS,GATA3,GCC2,GCLM,GFPT2,GFRA1,GGH,GHDC,GLOD4,GLRX3,GLS,GLYATL2,GMPR2,GMPS,GNA13,GNPDA2,GOLGA1,GOLPH3L,GP2,GPATCH1,GPD1L,GPT2,GREB1,GRHL1,GSTM3,GSTO2,GTF2I,GTPBP2,HAUS6,HAUS8,HDGFRP3,HECTD4,HEXIM1,HID1,HIF1AN,HK2,HK3,HMCES,HMCN1,HMGB3,HMGCL,HMGCS2,HNMT,HOMER1,HSD17B8,HSD3B7,HSP90AB1,HSPA14,HSPA2,HSPA4,HSPA5,HSPB1,HYOU1,ICAM1,IDO1,IFRD1,IFT140,IFT172,IFT43,IFT46,IFT74,IFT81,IGF2BP2,IGFBP4,IL6ST,IMP3,IMPA2,INCENP,INPP4B,IQGAP3,IQSEC1,IRS1,ISG20,ITGA6,IVD,KANK1,KARS,KATNA1,KCTD6,KCTD7,KDM3A,KERA,KHDRBS3,KIAA0020,KIAA1244,KIAA1598,KIF11,KIF13A,KIF13B,KIF14,KIF15,KIF16B,KIF18B,KIF22,KIF23,KIF2C,KIF3A,KIF4A,KIFC1,KPNA2,KRT16,KRT18,KRT19,KRT23,KRT6B,KRT8,KRT81,KTN1,LAMP3,LARP4B,LCN2,LCP1,LDHB,LGALS8,LGALSL,LGMN,LIMA1,LIMK2,LLGL2,LMX1B,LNPEP,LPIN1,LRBA,LRPPRC,LRRC17,LSM14B,LYN,LYPLAL1,LYRM9,LZTFL1,MAD2L1,MAGED2,MAGED4,MAN2B1,MAPK9,MAPRE2,MAPT,MASTL,MCAM,MCCC2,MCM2,MCM3,MCM4,MCM5,MCM6,MCM7,MDN1,MDP1,ME2,MEX3A,MFAP4,MICAL3,MICALL1,MIEN1,MISP,MKI67,MKL2,MKLN1,MLKL,MLPH,MMACHC,MMP1,MMP9,MOGS,MORC2,MPHOSPH10,MPI,MPP6,MPP7,MSH2,MSH6,MSI2,MSLN,MTHFD1L,MTHFD2,MVD,MYEOV2,MYO1D,MYO1E,MYO5C,MYO6,MYOF,N4BP3,NAMPT,NANS,NARFL,NAT1,NCAM2,NCAPD2,NCAPG,NCAPH,NCF2,NCK2,NCS1,NDC80,NDRG1,NDRG2,NEDD4,NEDD4L,NEK9,NFIB,NHSL1,NIP7,NME3,NOL10,NOL11,NOS1AP,NOSTRIN,NPEPPS,NSF,NT5C,NT5C2,NT5DC2,NUBPL,NUDCD1,NUDT12,NUDT16L1,NUDT3,NUF2,NUMA1,NXF1,OMD,OSCP1,OTUD7B,OXCT1,PAAF1,PADI2,PAFAH1B3,PAK1IP1,PARD6B,PATL1,PBK,PCBD2,PCBP4,PDCD6IP,PDE4DIP,PDGFRB,PDIA6,PDK1,PDZK1,PFAS,PGD,PGGT1B,PGPEP1,PGR,PHGDH,PHPT1,PHYHD1,PIK3AP1,PIR,PKIB,PLA2G16,PLA2G4A,PLAUR,PLCG2,PLCH1,PLEK,PLEKHF2,PLEKHG1,PLIN2,PLOD1,PLOD3,PLS1,PM20D2,PNP,PNPO,POLA1,POLA2,POLD2,POLR1B,PPP1R14C,PPP2R3A,PPP2R4,PPP2R5D,PRDX4,PREX1,PROM1,PROSER1,PRR15,PRRC1,PRTFDC1,PSAT1,PSME4,PSMG2,PTBP2,PTER,PTGR2,PTPLAD1,PTPN2,PTPN23,PTX3,PURA,PUS7,QDPR,RAB12,RAB27B,RABEP1,RABL3,RABL6,RALGPS2,RANGAP1,RAP1GDS1,RARA,RARRES1,RASAL1,RASEF,RASSF5,RBKS,RBM47,RCC2,RCN2,RCOR3,RDH10,RECQL4,REPS2,RERG,RFC2,RFC4,RHEB,RIF1,RING1,RIT1,RNGTT,RNPEP,RPLP2,RPS13,RPS2,RPS3,RRP7A,RTKN,RUFY2,RUNDC1,S100A13,SCCPDH,SCGB1D2,SCRN2,SCUBE2,SDF2,SEC14L2,SEC16A,SEC23IP,SEC63,SEMA3C,SEPSECS,SERPINB10,SFRP1,SFXN2,SFXN5,SGCD,SGSM3,SH3BGRL,

SH3BP1,SH3GLB2,SHMT2,SHPK,SIGIRR,SKIV2L,SLC25A13,SLC25A5,SLC27A2,SLC2A1,SLC3A2,SLC9A3R1,SMARCC2,SMC2,SMC4,SMCHD1,SND1,SNF8,SNX27,SORD,SOX10,SPATA20,SPC24,SPR,SRA1,SRC,SRPK1,SSC5D,SSH3,STARD10,STC2,STEAP3,STK3,STK39,STON2,STRN3,SUOX,SYAP1,SYBU,SYNCRIP,SYTL2,SYTL4,TACC3,TANC2,TARBP1,TATDN1,TBC1D17,TBC1D9,TBC1D9B,TC2N,TCEA3,TEAD3,TFF1,TFF3,THBS4,THEMIS2,THSD4,THTPA,TIA1,TINAGL1,TJP3,TLDC1,TLE3,TMEM132A,TMF1,TOM1L1,TOP2A,TP53BP1,TP53BP2,TP53RK,TPRG1,TPRN,TPSB2,TPX2,TRIM2,TRIM29,TRIM3,TRIO,TRMT6,TRMT61A,TSPYL5,TSTD1,TTC13,TTC26,TTC37,TTC39A,TTC8,TTK,TXNDC12,TYMS,UBASH3B,UBE2S,UBE2T,UBE3A,UBR1,UBTD2,UGDH,UGP2,UPF2,UPP1,USP47,VAC14,VAV3,WARS,WDHD1,WDR12,WDR19,WDR4,WDR74,WFS1,WNK4,XPC,XPNPEP1,XPO5,YES1,ZNF385A,ZNF446,ZNF552,ZPR1,ZWINT

Supplementary Data 2: List of differentially expressed proteins between Luminal A and HER2 positive subtype.

ABAT,ACADSB,ACOX1,AGO3,AGR3,AHSA1,AIF1L,AIM1,AKR1B10,ALDH18A1,AMACR,ANKRD17,APPL1,ASNS,ATP6V1G2,BDH2,BEND3,BOP1,BTAF1,C2CD5,C2orf54,C9orf64,CA12,CASP3,CCDC134,CDK1,CHORDC1,CKAP2,CKS1B,CLUH,COPG1,CROCC,CRYZ,DDB2,DDX39A,DDX41,DESI1,DNMT3A,DPP3,DYNLT1,EEF1G,EEF2,EMC1,EML2,ERBB2,ERO1L,ESR1,ESRP1,FAM114A2,FAM203A,FN3K,FOXRED1,G6PD,GGCT,GLOD4,GLRX3,GLYATL2,GMPS,GRB7,GREB1,GSDMA,GSK3B,HDAC5,HDLBP,HEATR3,HMGCS1,HSP90AA1,HSP90AB1,HSPA5,HYOU1,IDE,IDH2,IDI1,IFT74,IGFBP4,IL6ST,IMPDH1,INPPL1,IPO4,IRS1,KCTD15,KIF21A,KPNA2,KPNA4,KYNU,LEF1,LGMN,LMNA,LMNB2,LRPPRC,LSM14B,LSS,LYRM9,MAN2B1,MAPT,METTL1,MICAL3,MIEN1,MORC2,MOV10,MTHFD2,MVD,MVK,MYOF,NAA40,NAMPT,NCAPD2,NIP7,NOL10,NOL6,NPEPPS,NRD1,NT5DC2,OGT,OSBPL2,PABPC1,PAXBP1,PCNA,PGR,PHGDH,PHYHD1,PNMT,POLA2,PPP1R14B,PPP2R2A,PRDX4,PSME4,PSMG1,PURA,PYCR1,RAB3D,RABEP1,RANGAP1,RCOR3,REPS2,RRM2,S100A13,SART3,SCUBE2,SCYL1,SHMT2,SLC25A5,SMC2,SND1,STARD3,STAU1,STC2,SYNCRIP,SYTL4,TARS,THSD4,TOR3A,TOX4,TXNDC12,UBE2C,UBE2S,UBE2T,UGGT1,WDR12,WDR19,WDR26,XPNPEP3,XPO5

Supplementary Data 3: Comma separated list of known transcription regulatory interactions between proteins which were differentially expressed between Luminal A and Basal subtypes. Here X->Y means X is a known transcription regulator of Y according to HTRIDb database.

AR->ACTN4,AR->PLIN2,AR->AKT2,AR->APBB2,AR->AR,AR->ARL3,AR->ASS1,AR->BCL2,AR->CA12,AR->CALR,AR->CALU,AR->CBFB,AR->LRBA,AR->CDC20,AR->CDH3,AR->CDK6,AR->CENPE,AR->CENPF,AR->CTSC,AR->CKS1B,AR->CPA3,AR->CRYZ,AR->CST3,AR->CYB5A,AR->DIAPH2,AR->DLG3,AR->DMD,AR->DNMT3A,AR->DOCK1,AR->ECT2,AR->EEF1G,AR->EGFR,AR->ESR1,AR->ETFDH,AR->EXTL2,AR->EZH2,AR->FANCD2,AR->ACSL4,AR->FAT1,AR->FHIT,AR->FLNB,AR->FUT8,AR->GFRA1,AR->GCLM,AR->GLS,AR->ARHGAP35,AR->GTF2I,AR->HK2,AR->HNMT,AR->HSPA5,AR->IL6ST,AR-

>IMPA2,AR->ITGA6,AR->IRS1,AR->KPNA2,AR->KRT81,AR->KTN1,AR-
>LGALS8,AR->LYN,AR->MAD2L1,AR->MCM6,AR->ME2,AR->MYO1D,AR-
>MYO1E,AR->MYO6,AR->NCAM2,AR->NEDD4,AR->NFIB,AR->NUMA1,AR-
>OMD,AR->OXCT1,AR->PDZK1,AR->ATP8B1,AR->PGD,AR->PLCG2,AR-
>PLOD1,AR->POLA1,AR->PPP2R3A,AR->PPP2R4,AR->PKIB,AR->DNAJC3,AR-
>LGMN,AR->PTPN2,AR->PTX3,AR->RANGAP1,AR->RAP1GDS1,AR->RARA,AR-
>RARRES1,AR->RCN2,AR->RFC4,AR->RIT1,AR->RTKN,AR->S100A13,AR-
>SGCD,AR->SH3BGRL,AR->SLC2A1,AR->SORD,AR->TCEA3,AR->DYNLT1,AR-
>TIA1,AR->TLE3,AR->TP53BP1,AR->TP53BP2,AR->TRIO,AR->UBE3A,AR-
>UGDH,AR->UGP2,AR->UPP1,AR->XPC,AR->XPNPEP1,AR->DNALI1,AR-
>ANXA9,AR->NCK2,AR->CUL3,AR->BCAS1,AR->STC2,AR->RNGTT,AR-
>INPP4B,AR->CDC123,AR->AP3D1,AR->PLOD3,AR->ARHGEF2,AR->REPS2,AR-
>DHRS3,AR->PTER,AR->KIF23,AR->NPEPPS,AR->GCC2,AR->PDE4DIP,AR-
>NOS1AP,AR->DLGAP5,AR->CEP170,AR->KIF14,AR->KIAA0020,AR->GFPT2,AR-
>PDCD6IP,AR->TOM1L1,AR->SMC4,AR->SLC25A13,AR->LRRC17,AR->NDC80,AR-
>VAV3,AR->ECI2,AR->CAP2,AR->SYNCRIP,AR->SEMA3C,AR->IGF2BP2,AR-
>KHDRBS3,AR->GNA13,AR->AHCYL1,AR->MTHFD2,AR->FAM114A2,AR-
>EHD1,AR->MAPRE2,AR->SLC27A2,AR->CIT,AR->NUDT3,AR->WDHD1,AR-
>GALNT6,AR->SEC63,AR->PADI2,AR->TPX2,AR->NT5C2,AR->ERP44,AR-
>TBC1D9,AR->GPD1L,AR->LPIN1,AR->KANK1,AR->MDN1,AR->PSME4,AR-
>EHBP1,AR->KIF13B,AR->TRIM2,AR->NEDD4L,AR->SMCHD1,AR->NCAPH,AR-
>NCS1,AR->SEC14L2,AR->DNPEP,AR->SH3BP1,AR->POLA2,AR->MTHFD1L,AR-
>ZNF385A,AR->ANKRD17,AR->TANC2,AR->MYOF,AR->SND1,AR->FOXP1,AR-
>AHDC1,AR->STK39,AR->GRHL1,AR->EEF2K,AR->STRN3,AR->HDGFRP3,AR-
>TXNDC12,AR->SEPSECS,AR->DDX47,AR->FAM198B,AR->LIMA1,AR-
>PTPLAD1,AR->CXXC5,AR->TRMT6,AR->CYB5R1,AR->GALNT7,AR->RBM47,AR-
>PUS7,AR->EXOC6,AR->LZTFL1,AR->HAUS6,AR->BCAS3,AR->SYTL2,AR-
>RALGPS2,AR->ARHGAP17,AR->PNPO,AR->APPL2,AR->STEAP3,AR->IMP3,AR-
>KIF16B,AR->SYBU,AR->VAC14,AR->RCOR3,AR->WDR12,AR->FAM63A,AR-
>MYO5C,AR->PRTFDC1,AR->OTUD7B,AR->ANKRD50,AR->KIAA1244,AR-
>ERGIC1,AR->NHSL1,AR->PREX1,AR->EPB41L5,AR->BEND3,AR->KIAA1598,AR-
>WDR19,AR->SCUBE2,AR->PTBP2,AR->KIF13A,AR->RBKS,AR->CLSTN2,AR-
>MCCC2,AR->TINAGL1,AR->DNAJC1,AR->ATG3,AR->AHNAK,AR->MLPH,AR-
>FYCO1,AR->TTC13,AR->C6orf211,AR->AAGAB,AR->THSD4,AR->NOL10,AR-
>PAAF1,AR->DCAF11,AR->SNX27,AR->PPP1R14C,AR->NUF2,AR->DNAL1,AR-
>RASSF5,AR->HMCN1,AR->PARD6B,AR->CBR4,AR->UBASH3B,AR->RERG,AR-
>STON2,AR->NEK9,AR->HAUS8,AR->PIK3AP1,AR->SFXN2,AR->GSTO2,AR-
>TTC8,AR->CDYL2,AR->MSI2,AR->IQGAP3,AR->PM20D2,AR->MPP7,AR-
>SPC24,AR->BROX,AR->CDCA2,AR->RASEF,AR->MLKL,AR->RAB12,AR-
>C3orf58,AR->PHYHD1,AR->RABL3,AR->TPRG1,AR->C9orf142,CEBPB-
>ASNS,CEBPB->HSD17B8,ESR1->AARS,ESR1->PLIN2,ESR1->ARL3,ESR1-
>BAG1,ESR1->BCL2,ESR1->BCL2,ESR1->CA12,ESR1->CA12,ESR1->CDK1,ESR1-
>LRBA,ESR1->CDH3,ESR1->CDK6,ESR1->CEBPB,ESR1->EPHB3,ESR1->ESR1,ESR1-
>FBP1,ESR1->FUT8,ESR1->GATA3,ESR1->ARHGAP35,ESR1->HK2,ESR1-
>FOXA1,ESR1->HSPA4,ESR1->HSPA5,ESR1->IGFBP4,ESR1->IGFBP4,ESR1-
>ISG20,ESR1->KIF22,ESR1->KRT6B,ESR1->KRT8,ESR1->KRT19,ESR1-

>KRT19,ESR1->LCN2,ESR1->LGALS8,ESR1->LIMK2,ESR1->LYN,ESR1->MCM5,ESR1->MCM7,ESR1->MPI,ESR1->MYO1E,ESR1->NFIB,ESR1->PDZK1,ESR1->PDZK1,ESR1->PGR,ESR1->PGR,ESR1->PGR,ESR1->PGR,ESR1->PGR,ESR1->PGR,ESR1->PLS1,ESR1->POLD2,ESR1->PKIB,ESR1->PKIB,ESR1->RARA,ESR1->RARA,ESR1->RHEB,ESR1->SLC2A1,ESR1->SLC3A2,ESR1->TEAD3,ESR1->TFF1,ESR1->TFF1,ESR1->TFF1,ESR1->TFF1,ESR1->TFF3,ESR1->TFF3,ESR1->TOP2A,ESR1->TP53BP2,ESR1->TYMS,ESR1->CORO2A,ESR1->ANXA9,ESR1->BCAS1,ESR1->STC2,ESR1->STC2,ESR1->RABEP1,ESR1->DHRS3,ESR1->SLC9A3R1,ESR1->SLC9A3R1,ESR1->RECQL4,ESR1->PDE4DIP,ESR1->GREB1,ESR1->GREB1,ESR1->SEMA3C,ESR1->SEMA3C,ESR1->STARD10,ESR1->N4BP3,ESR1->TBC1D9,ESR1->DNPEP,ESR1->DNPEP,ESR1->ANKRD17,ESR1->TANC2,ESR1->MYOF,ESR1->FOXP1,ESR1->UBE2T,ESR1->EEF2K,ESR1->FAM198B,ESR1->CXXC5,ESR1->ANLN,ESR1->DDX49,ESR1->SYTL2,ESR1->ESRP1,ESR1->APPL2,ESR1->SYBU,ESR1->FAM63A,ESR1->RCC2,ESR1->MYO5C,ESR1->DNAJC12,ESR1->PREX1,ESR1->KIAA1598,ESR1->CLSTN2,ESR1->DSCC1,ESR1->THSD4,ESR1->NOL10,ESR1->HMCN1,ESR1->C9orf64,ESR1->RERG,ESR1->RERG,ESR1->UBTD2,ESR1->SYTL4,ESR1->AGR3,ESR1->UBR1,ESR1->FAM102A,ESR1->FAM102A,GATA3->PLIN2,GATA3->ASAH1,GATA3->CA12,GATA3->LRBA,GATA3->CDH3,GATA3->CDK6,GATA3->CETN3,GATA3->DLG3,GATA3->EGFR,GATA3->ERBB3,GATA3->FABP5,GATA3->FUT8,GATA3->GATA3,GATA3->GATA3,GATA3->ARHGAP35,GATA3->HK2,GATA3->HMGB3,GATA3->FOXA1,GATA3->IGFBP4,GATA3->ISG20,GATA3->KRT19,GATA3->LGALS8,GATA3->LIMK2,GATA3->LNPEP,GATA3->LYN,GATA3->MPI,GATA3->MYO1E,GATA3->MYO6,GATA3->PDZK1,GATA3->PGR,GATA3->PLS1,GATA3->PKIB,GATA3->RARA,GATA3->RING1,GATA3->SLC2A1,GATA3->SLC3A2,GATA3->FSCN1,GATA3->TARBP1,GATA3->TEAD3,GATA3->TFF1,GATA3->TFF3,GATA3->TP53BP2,GATA3->CORO2A,GATA3->XPC,GATA3->PIR,GATA3->STC2,GATA3->SLC9A3R1,GATA3->GREB1,GATA3->COL4A3BP,GATA3->DDX39A,GATA3->SEMA3C,GATA3->N4BP3,GATA3->TBC1D9,GATA3->DNPEP,GATA3->MMACHC,GATA3->ANKRD17,GATA3->TANC2,GATA3->EEF2K,GATA3->SCCPDH,GATA3->FAM198B,GATA3->LIMA1,GATA3->WDR74,GATA3->ESRP1,GATA3->APPL2,GATA3->GOLPH3L,GATA3->SYBU,GATA3->KDM3A,GATA3->DNAJC12,GATA3->ANKRD50,GATA3->EPB41L5,GATA3->KIAA1598,GATA3->CLSTN2,GATA3->C6orf211,GATA3->PLEKHF2,GATA3->NUBPL,GATA3->RASSF5,GATA3->HMCN1,GATA3->C9orf64,GATA3->MASTL,GATA3->RERG,GATA3->UBTD2,GATA3->TTC8,GATA3->TC2N,GATA3->RUNDC1,GATA3->AGR3,GATA3->UBR1,GATA3->FAM102A,FOXA1->BCL2,FOXA1->BCL2,FOXA1->CA12,FOXA1->LRBA,FOXA1->CDH3,FOXA1->CDK6,FOXA1->CETN3,FOXA1->EPHB3,FOXA1->FUT8,FOXA1->GATA3,FOXA1->ARHGAP35,FOXA1->HK2,FOXA1->FOXA1,FOXA1->IGFBP4,FOXA1->ISG20,FOXA1->KRT19,FOXA1->LGALS8,FOXA1->LNPEP,FOXA1->LYN,FOXA1->MCM7,FOXA1->MYO1E,FOXA1->MYO6,FOXA1->PDZK1,FOXA1->PGR,FOXA1->PLS1,FOXA1->PKIB,FOXA1->RARA,FOXA1->SLC2A1,FOXA1->TARBP1,FOXA1->TFF1,FOXA1->TFF3,FOXA1->TP53BP2,FOXA1->CORO2A,FOXA1->STC2,FOXA1->SLC9A3R1,FOXA1->GREB1,FOXA1->COL4A3BP,FOXA1->SEMA3C,FOXA1->N4BP3,FOXA1-

>TBC1D9,FOXA1->DNPEP,FOXA1->MMACHC,FOXA1->TANC2,FOXA1->SCCPDH,FOXA1->FAM198B,FOXA1->LIMA1,FOXA1->WDR74,FOXA1->ESRP1,FOXA1->APPL2,FOXA1->GOLPH3L,FOXA1->SYBU,FOXA1->MYO5C,FOXA1->DNAJC12,FOXA1->ANKRD50,FOXA1->EPB41L5,FOXA1->CLSTN2,FOXA1->NUBPL,FOXA1->HMCN1,FOXA1->MASTL,FOXA1->RERG,FOXA1->SFXN2,FOXA1->TTC8,FOXA1->TC2N,FOXA1->AGR3,FOXA1->FAM102A,PGR->BCL2,PGR->BCL2,PGR->ESR1,PURA->AR,SOX10->SOX10

Supplementary Data 4: Comma separated list of known transcription regulatory interactions between proteins which were differentially expressed between Luminal A and HER2 positive subtypes. Here X->Y means X is a known transcription regulator of Y according to HTRIDb database.

ESR1->CA12,ESR1->CA12,ESR1->CDK1,ESR1->ESR1,ESR1->G6PD,ESR1->GRB7,ESR1->HSPA5,ESR1->IGFBP4,ESR1->IGFBP4,ESR1->NRD1,ESR1->PGR,ESR1->PGR,ESR1->PGR,ESR1->PGR,ESR1->PGR,ESR1->PGR,ESR1->STC2,ESR1->STC2,ESR1->PSMG1,ESR1->KYNU,ESR1->RABEP1,ESR1->GREB1,ESR1->GREB1,ESR1->OSBPL2,ESR1->ANKRD17,ESR1->MYOF,ESR1->UBE2T,ESR1->ESRP1,ESR1->UGGT1,ESR1->NAA40,ESR1->THSD4,ESR1->C2orf54,ESR1->NOL10,ESR1->C9orf64,ESR1->LMNB2,ESR1->SYTL4,ESR1->AGR3,PGR->ESR1
